# Association of Post-Procedural Serum Sodium with 90-Day Mortality in Acute Ischaemic Stroke Patients Undergoing Mechanical Thrombectomy: A Retrospective Single-Centre Observational Study

**DOI:** 10.3390/diagnostics16172832

**Published:** 2026-09-02

**Authors:** Birgyl Kuki, Mehmet Yıldız, Şule Dalkılıç, Bilal Dalkılıç, Eren Kılıç, Semanur Aksu, Gülümser Büşranur Yurtsev Kaplan, Halil Alper Eryılmaz, Bilgehan Atılgan Acar

**Affiliations:** 1 Internal Medicine Clinic, Private Beyhekim Hospital, Sakarya 54100, Türkiye; birgyl.kuki@gmail.com; 2 Giresun Provincial Health Directorate, Bulancak Sehit Er Enver Erdogan Family Health Center, Giresun 28300, Türkiye; drmehmetyildiz54@gmail.com; 3 Department of Neurology, Akyazı State Hospital, Sakarya 54400, Türkiye; drsuledalkilic@gmail.com; 4 Department of Family Medicine, Faculty of Medicine, Sakarya University, Sakarya 54100, Türkiye; bilaldalkilic@sakarya.edu.tr; 5 Department of Neurology, Faculty of Medicine, Sakarya University, Sakarya 54100, Türkiyesemanuraksu54@gmail.com (S.A.); busranuryurtsev@gmail.com (G.B.Y.K.); 6 Department of Neurology, Sakarya Training and Research Hospital, Sakarya 54100, Türkiye; dr.alpereryilmaz@gmail.com

**Keywords:** electrolyte imbalance, endovascular procedures, ischemic stroke, prognosis, thrombectomy

## Abstract

**Background/Objectives**: Electrolyte disturbances are common in patients with Acute Ischemic Stroke (AIS) and may influence clinical outcomes. However, the prognostic significance of peri-procedural electrolyte changes in patients undergoing mechanical thrombectomy remains insufficiently investigated. This study aimed to evaluate the associations of pre- and post-procedural serum electrolyte levels and peri-procedural electrolyte changes with 90-day mortality in patients with AIS treated with mechanical thrombectomy. **Methods**: This retrospective observational study included 274 adult patients with anterior circulation large-vessel occlusion who underwent mechanical thrombectomy between February 2017 and February 2026. Serum sodium (Na), potassium (K), chloride (Cl), and calcium (Ca) levels were recorded before thrombectomy and within the first hour after the procedure. Peri-procedural electrolyte changes (Δ) were calculated as post-procedural minus pre-procedural values. Clinical, imaging, laboratory, and procedural variables associated with 90-day mortality were evaluated using univariate and multivariable logistic regression analyses. **Results**: Of the 274 patients, 67 (24.5%) died within 90 days. Post-procedural serum Na levels were significantly lower in the mortality group than in the survival group (*p* = 0.030). No significant associations were observed between 90-day mortality and pre-procedural electrolyte levels or peri-procedural electrolyte changes. In the multivariable analysis, higher admission National Institutes of Health Stroke Scale (NIHSS) scores (*p* = 0.001), symptomatic intracranial hemorrhage (*p* < 0.001), higher admission glucose levels (*p* = 0.003), and higher triglyceride levels (*p* = 0.046) were independently associated with increased 90-day mortality, whereas successful reperfusion (TICI 2b–3) (*p* = 0.011) and higher post-procedural serum Na levels (OR = 0.851, 95% CI: 0.749–0.968; *p* = 0.014) were independently associated with reduced odds of 90-day mortality. The addition of post-procedural serum Na to the base model resulted in a modest increase in discrimination (AUC 0.800 to 0.812; ΔAUC = 0.012), which was not statistically significant (DeLong *p* = 0.454). **Conclusions**: Among the peri-procedural electrolyte parameters evaluated, only post-procedural serum Na was independently associated with 90-day mortality after mechanical thrombectomy. However, adding post-procedural serum Na to established clinical, procedural, and metabolic predictors did not significantly improve model discrimination. These findings suggest that post-procedural serum Na may represent an associated prognostic marker, while its incremental predictive utility beyond established predictors remains unproven.

## 1. Introduction

Acute Ischemic Stroke (AIS) is one of the leading neurological diseases characterized by high morbidity and mortality rates worldwide, and it imposes a significant economic burden on healthcare systems and society [1]. A substantial increase in its incidence and prevalence has been observed over the past thirty years [2]. Particularly in cases of AIS resulting from large-vessel occlusion, mechanical thrombectomy (MT) has become the standard treatment approach, having been demonstrated to be safe and effective in numerous randomized controlled trials, and has led to significant improvements in functional outcomes [3].

However, despite the significant therapeutic benefits provided by MT, mortality remains a major clinical problem in AIS. In the HERMES meta-analysis, while the rate of good functional outcomes at three months was reported to be 46–51% in patients who underwent MT, the 6-month mortality rate was reported to be approximately 15% [4]. This suggests that successful recanalization does not always result in a life-saving outcome and that the mechanisms determining mortality following MT have not been fully elucidated. Cerebral hypoperfusion and hypoxia caused by persistent arterial occlusion can negatively affect the clinical course by causing the ischemic penumbra to progress to an irreversible infarct core [5].

Electrolytes play a critical role in fundamental physiological processes such as the maintenance of cellular functions, the preservation of osmotic and acid-base balance, and neuromuscular transmission. Among these electrolytes, sodium (Na) is the primary cation in extracellular fluid and is of fundamental importance for fluid balance and the maintenance of normal neurological function [6]. Electrolyte imbalances are commonly observed in patients with AIS. The most frequently encountered of these imbalances is hyponatremia, which is associated with cerebral edema, the development of seizures, and neurological deterioration [7]. In addition, hypokalemia, hypochloremia, and hypocalcemia are among the electrolyte imbalances frequently observed in clinical practice and can negatively affect patients’ clinical course and prognosis [8]. Since serum electrolyte levels may fluctuate during hospitalization, evaluating not only baseline values but also changes throughout the course of treatment may be important for prognosis.

While current clinical and imaging parameters provide important information for predicting the risk of 90-day mortality in patients with AIS who undergo MT, they do not, on their own, offer sufficient prognostic accuracy. Therefore, the identification of biomarkers that are easily obtainable in routine clinical practice, low-cost, and reliable constitutes one of the key focuses of current research in terms of early identification of high-risk patients, individualization of treatment strategies, and improvement of clinical outcomes. While serum electrolytes are among the promising biomarkers in this regard, studies evaluating the effect of pre-procedure and early post-procedure electrolyte levels as well as changes in these levels on 90-day mortality in patients with AIS undergoing MT remain limited. Therefore, this study investigated the association between serum electrolyte levels measured before and in the early post-procedure period, as well as changes in these levels, and 90-day mortality in patients undergoing MT for AIS.

## 2. Materials and Methods

### 2.1. Study Design, Setting, and Participants

This retrospective observational study included adult patients who underwent MT for AIS at the Neurology Clinic of Sakarya University Training and Research Hospital between 20 February 2017 and 20 February 2026. Demographic characteristics, clinical data, and laboratory parameters of the patients were retrospectively obtained from the hospital information management system and patient records. No additional interventional procedures, diagnostic evaluations, or treatments were performed as part of the study. All data were anonymized and processed in accordance with privacy principles.

Inclusion criteria were as follows: patients who presented to the Neurology Clinic at Sakarya Training and Research Hospital with AIS within the specified date range; patients aged 18 years and older; patients who underwent MT due to large vessel occlusion (LVO); the availability of pre-procedure serum electrolyte levels; and having control electrolyte levels measured within the first 0–1 h upon admission to the intensive care unit (ICU) following MT.

Exclusion criteria were as follows: patients under 18 years of age; those diagnosed with chronic kidney disease; those with posterior circulation occlusion; those undergoing hemodialysis; those with severe sepsis or septic shock; those with incomplete clinical or laboratory data; those without pre- or post-procedure electrolyte measurements; and those who received intravenous (IV) thrombolytic therapy.

Patients who received IV thrombolytic therapy were also excluded. In our centre, as part of routine clinical practice during the study period, these patients generally did not undergo routine post-procedural blood sampling immediately after MT, and follow-up laboratory measurements were instead obtained approximately 24 h after thrombolytic treatment. Consequently, post-procedural electrolyte measurements within the predefined 0–1 h window were unavailable for these patients. They were therefore excluded to maintain a consistent post-procedural sampling time across the study population and to avoid temporal measurement bias.

### 2.2. Sample Size of the Study

**Figure 1:**
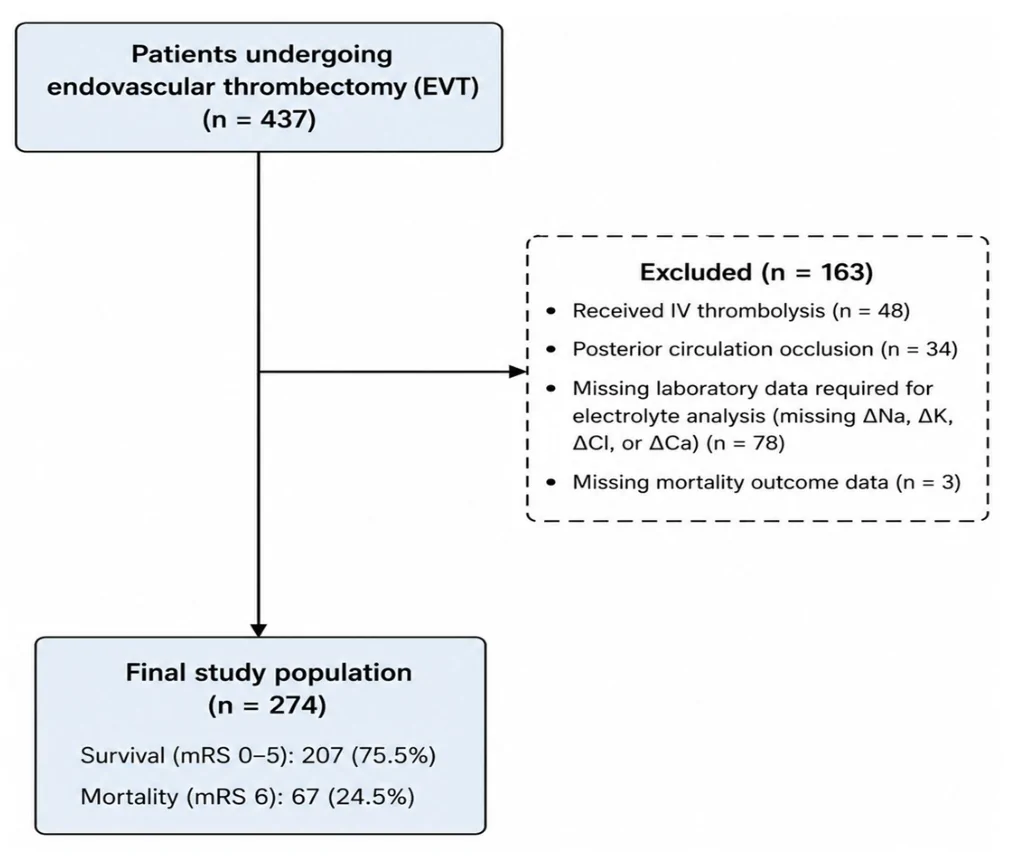
Flowchart of patient selection and final study population.

The study population consisted of a total of 437 adult patients who underwent MT at the Neurology Clinic during the study period following a diagnosis of AIS. After applying the inclusion and exclusion criteria, 274 patients with an appropriate data set were included in the study. The participant selection process is presented in Figure 1. Missing data were handled using a complete-case approach, and no statistical imputation was performed. Patients with incomplete laboratory data required for the primary analyses or missing 90-day mortality outcomes were excluded before the construction of the final analytical cohort. Of the 437 initially screened patients, 78 (17.8%) were excluded because of incomplete laboratory data and 3 (0.7%) because of missing 90-day mortality data. The final analytical cohort therefore consisted of patients with complete data for the variables required for the primary analyses.

This study employed a retrospective design, and the principle was to include all patients who met the eligibility criteria during the specified study period. For this reason, no sample size calculation was performed prior to the study. The research is based on a retrospective review of anonymized electronic medical records obtained during routine clinical practice. Laboratory parameters were measured and analyzed as part of the standard clinical care process, rather than within the framework of a predefined research protocol. A post hoc power analysis was subsequently performed to evaluate the statistical power of the study.

### 2.3. Data Collection

Demographic characteristics, clinical information, and laboratory results were obtained from the Neurology Clinic Information System, the Turkish Ministry of Health’s e-Nabız system, and the laboratory information management system. As part of the study, no questionnaires were administered to participants, no additional laboratory tests or biological sampling were performed, and no interventions beyond routine clinical practice were carried out. Prior to analysis, all patient records were anonymized, and personal identifying information was removed from the dataset before evaluation.

### 2.4. Variables

#### 2.4.1. Dependent Variable

The dependent variable of the study was defined as 90-day mortality status (yes/no). Functional outcome at 90 days was assessed using the Modified Rankin Scale (mRS). The 90-day mRS and vital status were ascertained during routine outpatient neurology follow-up visits or, when an in-person assessment was unavailable, through telephone interviews with the patient’s primary caregiver. Patients were categorized into two groups according to their 90-day outcomes: those who were alive at 90 days after stroke onset constituted the survival group (mRS 0–5), whereas patients who died within 90 days constituted the mortality group (mRS 6). Ninety-day mortality was used as the dependent variable in all logistic regression analyses.

#### 2.4.2. Basic Independent Variables

The primary independent variables of the study were serum electrolyte levels (Na, potassium [K], chloride [Cl], and calcium [Ca]) measured before the procedure and in the early post-procedure period, along with the delta (Δ) values indicating the peri-procedural changes in these parameters. Serum electrolyte levels were obtained from a blood sample drawn prior to MT at the time of presentation to the emergency department and from the first follow-up blood sample drawn within the first 0–1 h after admission to the ICU following MT. Peri-procedural electrolyte change was calculated by subtracting the pre-procedural electrolyte level from the post-procedural electrolyte level and was defined as Δ electrolyte (post-procedural electrolyte minus pre-procedural electrolyte). The association between pre-procedural and post-procedural serum electrolyte levels and peri-procedural electrolyte changes (Δ electrolyte) with 90-day mortality was evaluated.

The 0–1 h sampling window reflected the logistical time required for transfer from the angiography suite to the stroke ICU and initial stabilization. The post-procedural blood sample was obtained as part of the initial laboratory assessment immediately following ICU admission. Importantly, no additional intravenous fluids, contrast media, mannitol, or hypertonic saline were administered during the transfer period or after ICU admission before collection of the post-procedural blood sample. Thus, no additional fluid administration or therapeutic intervention occurred between completion of the procedure and post-procedural blood sampling. The study also examined age (years), gender (female/male), smoking, alcohol use, hypertension (HT), diabetes mellitus (DM), obesity, dyslipidemia, atrial fibrillation (AF), coronary artery disease (CAD), history of coronary artery bypass grafting (CABG), peripheral artery disease (PAD), history of malignancy, and history of stroke or transient ischemic attack (TIA); the need for intubation due to respiratory distress during the procedure; the presence of complications associated with endovascular therapy (EVT); and the development of symptomatic intracranial hemorrhage following EVT. These demographic and clinical variables were recorded as present or absent. Procedural variables including the time from symptom onset to reperfusion (minutes) and the status of successful reperfusion were assessed according to the Thrombolysis in Cerebral Infarction (TICI) classification. TICI 2b, 3 was defined as successful reperfusion, while TICI 0–2a was defined as unsuccessful reperfusion [9]. Stroke etiology was classified according to the Trial of Org 10172 in Acute Stroke Treatment (TOAST) classification as large artery atherosclerosis, cardioembolic, small vessel occlusion, other specified causes, and stroke of undetermined cause [10].

The severity of the stroke at the time of presentation was assessed using the National Institutes of Health Stroke Scale (NIHSS). The NIHSS is a standardized clinical measure that assesses the severity of neurological deficits and ranges from 0 to 42 points. Generally, a score of 0 indicates a normal neurological examination, 1–4 indicates mild, 5–15 indicates moderate, 16–20 indicates moderate-to-severe, and 21–42 indicates severe neurological deficits. In the analyses, the NIHSS score recorded at the time of presentation to the emergency department prior to mechanical thrombectomy was used [11]. The initial extent of the ischemic lesion was assessed using the Alberta Stroke Programme Early CT Score (ASPECTS). ASPECTS is a 10-point semiquantitative scoring system used to evaluate early ischemic changes in the middle cerebral artery territory on noncontrast cranial computed tomography (CT) images. The score ranges from 0 to 10, with higher scores indicating a smaller infarct core and lower scores indicating a larger infarct core. Clinically, ASPECTS of 8–10 are considered consistent with a minimal infarct core, 6–7 with a moderate infarct core, and ≤5 with a large infarct core [12].

Laboratory variables, including hemoglobin (g/dL), hematocrit (%), white blood cells (×10^9^/L), neutrophils (×10^9^/L), lymphocytes (×10^9^/L), monocytes (×10^9^/L), platelets (×10^9^/L), glucose (mg/dL), total cholesterol (mg/dL), low-density lipoprotein cholesterol (LDL-C, mg/dL), high-density lipoprotein cholesterol (HDL-C, mg/dL), triglycerides (mg/dL), and albumin (g/dL) were recorded.

In multivariate logistic regression analyses, variables found to be clinically significant and statistically appropriate in univariate analyses were included in the model as covariates to assess the independent association between serum electrolyte levels and peri-procedural electrolyte changes and 90-day mortality.

### 2.5. Statistical Analysis

All statistical analyses were performed using IBM SPSS Statistics for Windows, version 26.0 (IBM Corp., Armonk, NY, USA). The normality of continuous variables was assessed using visual methods such as histograms and Quantile–Quantile (Q–Q) plots, as well as the Kolmogorov–Smirnov and Shapiro–Wilk tests when appropriate. Continuous variables that followed a normal distribution were presented as mean ± standard deviation (SD), while those that did not follow a normal distribution were presented as median and interquartile range (IQR). Categorical variables were expressed as frequency (*n*) and percentage (%). In comparisons between the survival (mRS 0–5) and mortality (mRS 6) groups, categorical variables were analyzed using the Pearson chi-square test or Fisher’s exact test when appropriate. For comparisons of continuous variables, the independent-samples *t*-test was used for normally distributed data, and the Mann–Whitney U test was used for non-normally distributed data. Stroke etiology was classified into four groups: large-artery atherosclerosis, cardioembolism, other specified etiologies, and unspecified etiologies. The distribution of stroke etiologies between the survival and mortality groups was compared using the chi-square test. Peri-procedural electrolyte parameters were evaluated as pre-procedural, post-procedural, and change (Δ) values. Univariate logistic regression analyses were performed for clinically significant demographic, procedural, laboratory, and electrolyte variables to identify factors associated with mortality. These univariate analyses, including comparisons across the peri-procedural electrolyte parameters, were considered exploratory and hypothesis-generating; therefore, no formal adjustment for multiple comparisons was applied. Accordingly, isolated findings from the univariate analyses were interpreted cautiously and were not considered confirmatory evidence of independent associations. The primary interpretation focused on associations evaluated within the multivariable framework and their stability in bootstrap analyses. Covariates for the multivariable models were selected based on clinical relevance and their established association with outcomes after AIS rather than solely on statistical significance in the univariate analyses. Three multivariable logistic regression models were constructed to evaluate the independent associations of pre-procedural sodium, post-procedural sodium, and peri-procedural sodium change (Δ sodium) with 90-day mortality. A consistent adjustment set comprising admission NIHSS score, successful reperfusion, intraprocedural intubation due to respiratory distress, symptomatic intracranial hemorrhage following EVT, admission glucose, triglyceride levels, and symptom onset-to-reperfusion time was used across all three models. Pre-procedural sodium, post-procedural sodium, and Δ sodium were entered into separate models because Δ sodium was mathematically derived from the pre- and post-procedural measurements, and simultaneous inclusion of these highly related parameters could introduce collinearity. Continuous variables were entered as continuous measures. Odds ratios (ORs) were reported per 1-unit increase for admission NIHSS (1 point), glucose (1 mg/dL), and sodium parameters (1 mmol/L), whereas the OR for triglycerides was expressed per 10 mg/dL increase to provide a more clinically interpretable effect estimate. Results were reported as ORs and 95% confidence intervals (CIs). In all analyses, a two-sided *p*-value < 0.05 was considered statistically significant.

Given the limited number of mortality events relative to the number of covariates, the stability of all three multivariable models was further assessed using nonparametric bootstrap resampling with 2000 samples. Bias-corrected and accelerated (BCa) 95% CIs were calculated for the regression coefficients.

To assess the incremental prognostic value of post-procedural serum Na, receiver operating characteristic (ROC) curve analysis was performed using predicted probabilities derived from two nested logistic regression models. The base model included admission NIHSS score, successful reperfusion, intraprocedural intubation due to respiratory distress, symptomatic intracranial hemorrhage following EVT, admission glucose, triglyceride levels, and symptom onset-to-reperfusion time, whereas the extended model additionally included post-procedural serum Na. Model discrimination was quantified using the area under the ROC curve (AUC) with 95% CIs, and the AUCs of the two correlated models were compared using DeLong’s test. Calibration of the extended model was assessed using the Hosmer–Lemeshow goodness-of-fit test and visually examined using a calibration plot comparing observed and predicted 90-day mortality probabilities across deciles of predicted risk.

A post hoc power analysis was performed using G*Power version 3.1.9.7 based on the observed difference in post-procedural serum sodium levels between the survival and mortality groups. Using a two-sided independent-samples *t*-test, an effect size of Cohen’s d = 0.300, an alpha level of 0.05, and group sizes of 207 and 67, the achieved statistical power (1 − β) was 56.7%.

### 2.6. Use of Generative Artificial Intelligence

A generative artificial intelligence tool (ChatGPT-5.6 Sol, OpenAI) was used during manuscript preparation to assist with language refinement and the drafting and revision of selected textual content. It was not used for study design, data collection, data generation, or statistical analysis. All AI-assisted content was critically reviewed, verified, and edited by the authors, who take full responsibility for the final manuscript.

## 3. Results

### 3.1. Demographic Characteristics of the Participants

Of the 274 patients included in the study, 207 (75.5%) were in the survival group and 67 (24.5%) were in the mortality group. No significant differences were found between the survival and mortality groups in terms of age, sex, smoking, chronic alcohol use, HT, DM, obesity, dyslipidemia, AF, CAD, PAD, history of malignancy, and previous stroke/TIA (*p* > 0.05). In contrast, a history of CABG was found to be significantly more common in the mortality group compared to the survival group (11.9% vs. 4.8%; *p* = 0.049) (Table 1).

### 3.2. Distribution of Stroke Etiologies by Clinical Outcome

Table 2 compares the distribution of stroke etiologies. Large-artery atherosclerosis accounted for 14.6% (30) in the survival group and 17.9% (12) in the mortality group; cardioembolic stroke accounted for 56.8% (117) and 46.3% (31); other identified etiologies accounted for 4.9% (10) and 13.4% (9), respectively; and undetermined etiology was found in 23.8% (49) and 22.4% (15) of cases, respectively. However, no statistically significant difference was found between the survival and mortality groups in terms of the distribution of stroke etiologies (*p* = 0.079) (Table 2).

### 3.3. Comparison of Procedural and Imaging Findings by Mortality

Table 3 compares the procedural and imaging characteristics of the mortality and survival groups. No significant differences were found between the groups in terms of systolic blood pressure (SBP) at presentation, diastolic blood pressure (DBP) at presentation, time from symptom onset to reperfusion, or complications associated with EVT (*p* > 0.05). In contrast, the admission NIHSS score was found to be significantly higher in the mortality group compared to the survival group (19 [16–20] vs. 17 [14–18]; *p* < 0.001). The ASPECTS at presentation was found to be significantly lower in the mortality group (9 [8–9] vs. 9 [8–10]; *p* = 0.039). The rate of TICI 2b, 3 was significantly higher in the survival group than in the mortality group (90.8% vs. 76.1%; *p* = 0.001). The need for intubation due to respiratory distress during the procedure was significantly more common in the mortality group compared to the survival group (9.0% vs. 1.0%; *p* = 0.003). Furthermore, the incidence of symptomatic intracranial hemorrhage following EVT was significantly higher in the mortality group than in the survival group (31.3% vs. 8.2%; *p* < 0.001) (Table 3).

### 3.4. Hematological and Biochemical Characteristics of the Survival and Mortality Groups

Table 4 compares the hematological and biochemical parameters. The baseline glucose level was 123.0 (103.0–151.0) mg/dL in the survival group and 164.0 (124.0–201.0) mg/dL in the mortality group and was significantly higher in the mortality group (*p* < 0.001). Similarly, triglyceride levels were 73.0 (56.0–110.0) mg/dL in the survival group and 99.5 (70.0–131.0) mg/dL in the mortality group, and the difference between the groups was statistically significant (*p* = 0.001). In contrast, no significant differences were observed between the groups in terms of hemoglobin, hematocrit, white blood cell count, platelet count, total cholesterol, LDLC, HDLC, and albumin levels (*p* = 0.530) (Table 4).

### 3.5. Comparison of Peri-Procedural Electrolyte Parameters According to Clinical Outcome

Pre-procedural and post-procedural electrolyte parameters were compared between the mortality and survival groups. The post-procedure serum Na level was found to be 136.36 ± 2.56 mmol/L in the survival group and 135.55 ± 2.83 mmol/L in the mortality group; the difference between the groups was statistically significant (*p* = 0.030). In contrast, no significant differences were observed between the groups in pre-procedure Na, K, Cl, and Ca levels, or in post-procedure Cl, K, and Ca levels (*p* > 0.05). Similarly, no statistically significant differences were observed between the groups in the electrolyte change values (ΔNa, ΔK^+^, ΔCl^−^, and ΔCa^2+^) (*p* > 0.05) (Table 5).

### 3.6. Univariate Logistic Regression Analysis of Mortality Predictors

A history of prior CABG increased the risk of mortality by 2.67 times (OR: 2.671, 95% CI: 1.008–7.076; *p* = 0.049). Each 1-point increase in the baseline admission NIHSS score increased the likelihood of mortality by a factor of 1.20 (OR: 1.199, 95% CI: 1.095–1.313; *p* < 0.001). The need for intubation due to respiratory distress during the procedure (OR: 10.033, 95% CI: 1.974–50.984; *p* = 0.003), symptomatic intracranial hemorrhage after EVT (OR: 5.102, 95% CI: 2.494–10. 440; *p* < 0.001), glucose level at presentation (OR: 1.008, 95% CI: 1.004–1.012; *p* < 0.001), and triglyceride level (OR: 1.050, 95% CI: 1.013–1.089; *p* = 0.001) showed a significant positive association with mortality. In contrast, successful reperfusion (OR: 0.288, 95% CI: 0.136–0.610; *p* = 0.001) and post-procedure serum Na level (OR: 0.890, 95% CI: 0.800–0.990; *p* = 0.031) were inversely associated with mortality. No statistically significant association with mortality was found for other clinical variables, including CAD, ASPECTS, pre-procedural Na, peri-procedural K, peri-procedural Cl, peri-procedural Ca, and electrolyte change values (all *p* > 0.05) (Table 6).

### 3.7. Multivariate Logistic Regression Analysis of Mortality Predictors

After multivariable adjustment, pre-procedural Na was not independently associated with mortality (OR: 0.917, 95% CI: 0.821–1.024; *p* = 0.124), whereas higher post-procedural sodium remained independently associated with lower odds of mortality (OR: 0.851, 95% CI: 0.749–0.968; *p* = 0.014). Each 1 mmol/L increase in post-procedural sodium was associated with an approximately 15% reduction in the odds of mortality. Δ sodium was not independently associated with mortality (OR: 0.939, 95% CI: 0.819–1.076; *p* = 0.364). Across all three models, admission NIHSS, successful reperfusion, intraprocedural intubation, symptomatic intracranial hemorrhage, glucose, and triglyceride levels remained independently associated with mortality. Symptom onset-to-reperfusion time was not statistically significant in any of the three models (all *p* > 0.05) (Table 7).

The stability of the three multivariable models was further assessed using 2000-sample nonparametric bootstrap resampling. In the model evaluating post-procedural serum Na, its inverse association with 90-day mortality remained statistically significant after bootstrap validation (bootstrap *p* = 0.023; BCa 95% CI for B: −0.312 to −0.045). In contrast, pre-procedural serum Na (bootstrap *p* = 0.133; BCa 95% CI for B: −0.212 to 0.038) and ΔNa (bootstrap *p* = 0.389; BCa 95% CI for B: −0.213 to 0.062) remained non-significant. These findings supported the stability of the principal association observed for post-procedural serum Na (Table 8).

To assess the incremental discriminatory value of post-procedural serum Na, the performance of the base model was compared with that of an extended model, additionally including post-procedural Na. The base model yielded an AUC of 0.800 (95% CI: 0.738–0.862), whereas the extended model, additionally including post-procedural serum Na, yielded an AUC of 0.812 (95% CI: 0.752–0.871). Although the AUC increased numerically by 0.012 following the addition of post-procedural serum Na, the difference between the two correlated ROC curves was not statistically significant (DeLong test, *p* = 0.454). Thus, although post-procedural serum Na remained independently associated with 90-day mortality, its addition did not significantly improve model discrimination beyond the established clinical, procedural, and metabolic predictors included in the base model.

**Figure 2:**
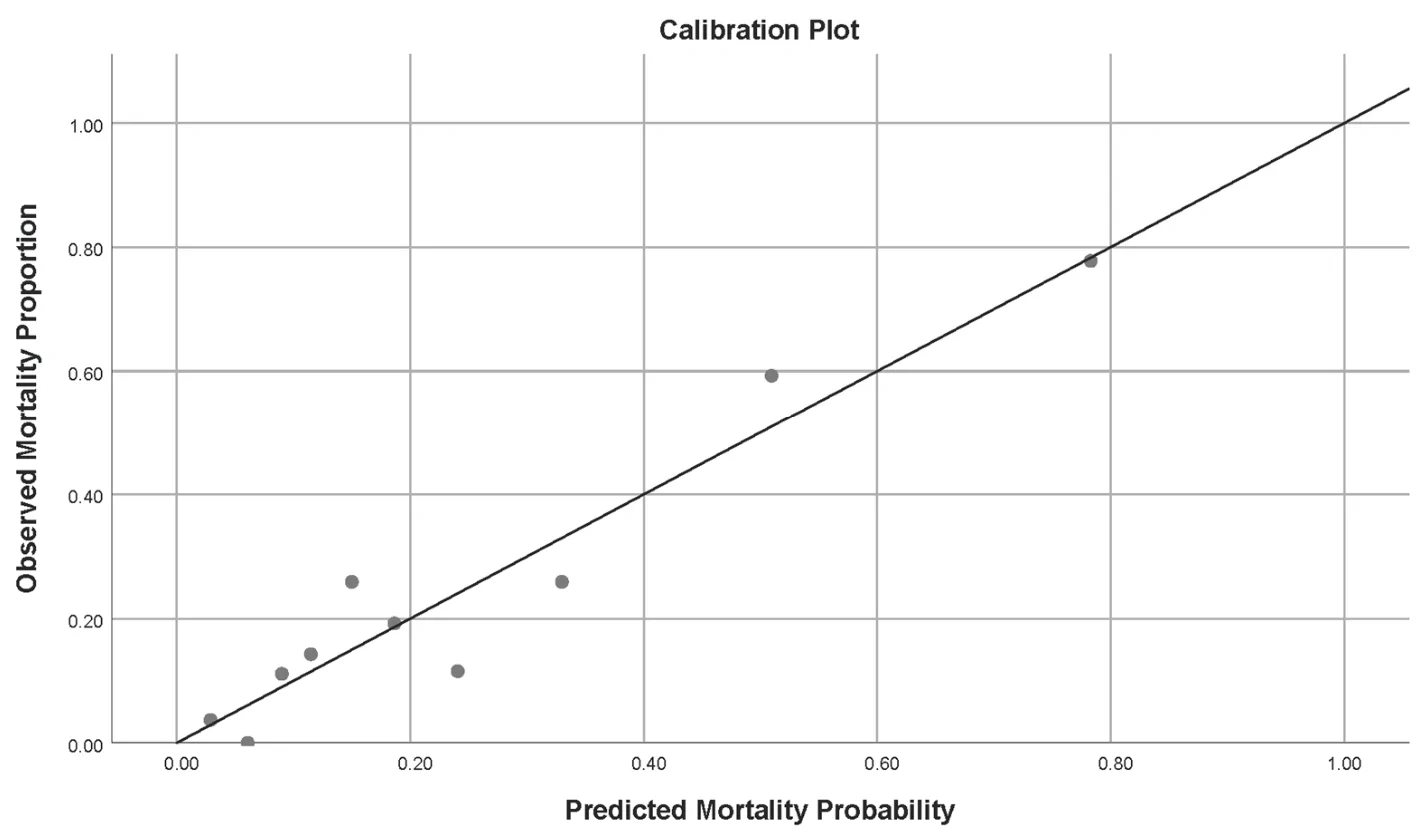
Calibration plot of the multivariable model including post-procedural serum sodium for 90-day mortality. Footnotes: Observed mortality proportions are plotted against mean predicted mortality probabilities across deciles of predicted risk. The diagonal line represents perfect agreement between observed and predicted probabilities.

The extended model also showed no evidence of poor calibration according to the Hosmer–Lemeshow goodness-of-fit test (χ^2^ = 7.159, df = 8, *p* = 0.520). Calibration was additionally examined graphically by comparing observed and predicted 90-day mortality probabilities across deciles of predicted risk (Figure 2).

## 4. Discussion

In our study, we evaluated the relationship between pre- and post-procedural serum electrolyte levels, peri-procedural changes in these levels, and 90-day mortality in patients who underwent MT for AIS. Our findings indicate that post-procedural serum Na levels were independently associated with 90-day mortality. In contrast, peri-procedural K, Cl, and Ca levels, as well as changes in these electrolytes, were not independently associated with 90-day mortality.

Post-procedural serum Na levels were independently and inversely associated with 90-day mortality. In the multivariable logistic regression model including post-procedural sodium, each 1 mmol/L increase in post-procedural serum Na was associated with an approximately 15% reduction in the odds of mortality (Table 7). These findings suggest that early post-procedural serum Na may serve as a prognostic marker of 90-day mortality after MT. Our findings are consistent with the literature. Pelouto et al. (2024) reported that three-month functional outcomes were worse in patients with hyponatremia compared to normonatremic patients, and that hyponatremia may independently serve as a marker of poor functional outcomes and 90-day mortality [13]. Similarly, Refardt et al. (2021) demonstrated an association between hyponatremia and increased mortality; however, they noted that a definitive conclusion could not be reached regarding whether this association was directly causal or merely a marker reflecting disease severity [14]. When considered together with the existing literature, our findings raise the hypothesis that low post-procedural serum Na may reflect pathophysiological processes associated with secondary brain injury following AIS rather than simply representing an isolated laboratory abnormality. However, the present observational data cannot establish whether Na disturbances contribute directly to secondary brain injury or merely reflect greater underlying disease severity. From a pathophysiological perspective, hyponatremia has been associated with osmotic and ionic disturbances that may promote cerebral edema, disruption of blood–brain barrier integrity, increased intracranial pressure, and reduced cerebral perfusion [15,16,17]. These mechanisms could theoretically be relevant to the progression of ischemic injury and adverse clinical outcomes; however, they were not directly evaluated in the present study and therefore should not be interpreted as mechanisms underlying the observed association between post-procedural serum Na and mortality. Although post-procedural serum Na remained independently associated with 90-day mortality, the model performance analysis showed that its addition to established clinical, procedural, and metabolic predictors resulted in only a modest numerical increase in discrimination (AUC 0.800 to 0.812; ΔAUC = 0.012), which was not statistically significant (DeLong *p* = 0.454). The extended model showed no evidence of poor calibration according to the Hosmer–Lemeshow goodness-of-fit test (χ^2^ = 7.159, df = 8, *p* = 0.520), with calibration also assessed graphically across deciles of predicted risk. Therefore, the present findings do not demonstrate significant incremental predictive utility of post-procedural serum Na beyond the predictors included in the base model. However, our findings do not establish that maintaining or correcting serum Na levels improves clinical outcomes. In addition, it is important to note that our study did not measure specific mechanistic markers such as cerebral edema volume, blood–brain barrier integrity, or inflammatory cytokines. Therefore, the proposed pathophysiological mechanisms linking post-procedural sodium to secondary brain injury remain purely hypothetical and highlight an area for future mechanistic research.

One of the striking aspects of our findings is that, while post-procedure serum Na levels independently served as a marker of 90-day mortality, pre-procedure Na levels and peri-procedural Na change did not reach statistical significance. Although there are studies in the current literature examining the relationship between serum Na levels and the prognosis of AIS, the vast majority of these have focused on Na levels at the time of presentation; studies evaluating the prognostic significance of serum Na levels measured in the early period following EVT are quite limited. In this regard, our study is among the few to demonstrate an independent association between early post-procedural serum Na levels and 90-day mortality after MT. Rather than implying a causal effect, our findings suggest that post-procedural serum Na may serve as an easily accessible prognostic marker for early risk stratification in this population.

In AIS, hyponatremia is generally considered an indicator of an imbalance in water and Na levels, most often resulting from the syndrome of inappropriate antidiuretic hormone secretion or cerebral salt-wasting syndrome [18]. Hyponatremia can lead to increased intracranial pressure and reduced cerebral perfusion by exacerbating cerebral edema. Considering the pathophysiological mechanisms, numerous studies have reported that hyponatremia is associated with increased mortality, longer hospital stays, and poor functional outcomes in patients with AIS [19].

However, it cannot be said that low post-procedural serum Na levels are a direct cause of mortality. More likely, this condition may serve as a biochemical indicator of extensive infarct volume, more severe cerebral edema, disruption of the blood–brain barrier, and the accompanying neurohormonal responses. Therefore, serum Na can be considered an easily accessible prognostic marker that reflects the severity of the disease rather than the pathophysiological process itself. Prospective mechanistic studies are needed to confirm this possibility.

This is consistent with previous studies reporting that cardioembolism is the most common etiological cause in patients with vascular occlusion who undergo MT. In their study evaluating 1169 patients who underwent mechanical thrombectomy, Gürkaş et al. reported that cardioembolism was the most common etiology (45.5%) [20].

In our study, we found that the admission NIHSS score at presentation was higher and the ASPECTS was lower in the mortality group; conversely, the successful reperfusion rate was higher in the survival group, and the incidence of symptomatic intracranial hemorrhage following EVT was higher in the mortality group. No significant differences were observed between the groups in terms of SBP and DBP at presentation, the time from symptom onset to recanalization, or EVT-related complications. These findings are consistent with the literature. Abecassis et al. (2023) reported that an increase in the admission NIHSS score at presentation is one of the key predictors of mortality and poor functional outcome [21].

The finding in our study that the admission NIHSS score at presentation was significantly higher in the mortality group supports the established prognostic role of the severity of initial neurological deficit in AIS. Studies have shown that, among patients with large-core infarcts undergoing EVT, an increase in baseline admission NIHSS score is independently associated with 90-day mortality, and that each one-point increase in admission NIHSS is associated with a significantly increased risk of 90-day mortality [22]. Current literature demonstrates that the admission NIHSS score is not merely a clinical scale reflecting the severity of initial neurological deficit, but also an important prognostic indicator of mortality in patients with AIS. Consistent with these observations, our findings showed that each one-point increase in admission NIHSS was independently associated with higher odds of 90-day mortality in patients undergoing EVT [23]. There are numerous studies in the literature supporting the association between the initial NIHSS score and 90-day mortality. In a large population-based cohort study, it was reported that high NIHSS scores were strongly associated with 30-day mortality and that, particularly in patients with an initial NIHSS score above 16, the risk of 30-day death was approximately 15 times higher compared to those with an NIHSS score <9. These findings indicate that the NIHSS is not merely a clinical scale reflecting the severity of initial neurological deficit; they also support the view that it is an important prognostic marker for predicting short-term mortality [24]. A recent cohort study found that the baseline NIHSS score is a strong predictor of 90-day mortality, independent of pre-stroke clinical characteristics, and that higher NIHSS scores are linearly associated with an increased risk of mortality [25]. These findings, consistent with the results of our study, support the notion that a high baseline admission NIHSS score is one of the most important clinical indicators predicting 90-day mortality in AIS.

The finding that the ASPECTS was significantly lower in the mortality group than in the survival group is consistent with the literature. Numerous studies have shown that a low ASPECTS, which reflects a larger infarct core, is associated with increased mortality and poorer functional outcomes. In a study conducted by Kaesmacher et al. in patients with an ASPECTS ≤5, it was reported that EVT yielded better clinical outcomes compared to medical therapy, and that TICI 2b-3 was associated with better functional outcomes [26]. These data support the notion that a low ASPECTS is one of the key predictors of a poor prognosis.

The rate of successful reperfusion was significantly higher in the survival group. Previous studies have reported that successful reperfusion at the TICI 2b, 3 level is associated with greater functional independence, lower 90-day mortality, and better clinical outcomes. Furthermore, in a recent meta-analysis evaluating patients with AIS due to vascular occlusion, the rate of TICI 2b, 3 following MT was found to be approximately 80% [27]. Our findings are consistent with the previously reported association between successful reperfusion and more favourable outcomes.

Although earlier reperfusion is a universally established predictor of better outcomes, the time from symptom onset to reperfusion did not significantly differ between the survival and mortality groups in our cohort, potentially reflecting consistently timely interventions and standardized procedural workflows across our centre.

In our study, higher baseline triglyceride levels were significantly associated with 90-day mortality in both the univariate and multivariable analyses. This association remained significant after adjustment for admission NIHSS score, successful reperfusion, intraprocedural intubation, symptomatic intracranial hemorrhage, admission glucose, and the respective peri-procedural sodium parameter across all three multivariable models. The relationship between lipid profiles and outcomes after AIS is complex, and previous studies have reported nonlinear, including U-shaped, associations between triglyceride levels and clinical outcomes [28,29]. In our cohort, higher triglyceride levels may reflect an adverse underlying metabolic and vascular risk profile; however, the present observational data do not allow us to determine the mechanisms underlying this association. Therefore, triglyceride levels may represent an additional prognostic marker in patients undergoing MT, but this finding should be interpreted cautiously and requires confirmation in larger prospective studies.

One of the unique aspects of our study is that, in addition to the combined assessment of key electrolytes before and after the procedure, changes in electrolyte levels were also analyzed from a prognostic perspective. There are very few studies that systematically examine peri-procedural electrolyte changes in patients with AIS who undergo MT. Our findings suggest that, among the peri-procedural electrolyte parameters evaluated, only post-procedural serum Na was independently associated with 90-day mortality. This association should be interpreted as hypothesis-generating and does not establish a causal relationship between post-procedural Na levels and mortality. Residual confounding cannot be excluded despite multivariable adjustment.

From a clinical perspective, post-procedural serum Na is an easily accessible, low-cost, and routinely measured laboratory parameter. Its independent association with 90-day mortality suggests potential value as a prognostic marker; however, the absence of a statistically significant improvement in model discrimination after its addition to established predictors indicates that its incremental predictive utility remains unproven. Accordingly, post-procedural serum Na should not currently be considered an established tool for improving risk stratification beyond conventional clinical, procedural, and metabolic predictors. However, since the present study is observational in nature, it is not possible to draw any conclusions regarding whether correcting serum Na levels improves clinical outcomes. These findings suggest that the primary determinants of 90-day mortality in AIS are initial stroke severity, successful reperfusion, symptomatic intracranial hemorrhage, and post-procedural metabolic changes; in particular, post-procedural serum Na levels may serve as a biomarker that could contribute to prognostic assessment in the early stages. Post-procedural serum Na remained independently associated with 90-day mortality after adjustment for these established predictors; however, its addition did not significantly improve model discrimination. Accordingly, serum Na may be considered an associated prognostic marker, but the present data do not establish incremental predictive value beyond conventional predictors. However, these findings need to be confirmed by larger, multicenter, and prospective studies.

### Limitations

This study has several limitations that should be acknowledged. First, the relatively small sample size and retrospective, single-centre design limit the external validity of our findings, meaning that the results may not be universally applicable to centres with different patient demographics, practice patterns, or peri-procedural fluid management protocols. Furthermore, we excluded a substantial proportion of real-world patients, specifically those receiving bridging intravenous thrombolysis, because early post-procedural laboratory measurements within the predefined 0–1 h sampling window were not routinely available in these patients, with follow-up laboratory assessments generally obtained approximately 24 h after thrombolytic treatment. This exclusion further restricts the generalisability of our results to the broader AIS population. Patients with posterior circulation stroke were also excluded; consequently, our findings should be interpreted with caution when extrapolated to the broader AIS population. Validation in larger, multicentre, and more diverse populations is therefore required.

Second, serum electrolyte measurements were limited to the pre-procedural period and the first hour after MT; therefore, subsequent electrolyte fluctuations during hospitalization could not be evaluated.

Third, serum osmolality, urine Na, urine osmolality, and neuroendocrine markers were unavailable, preventing differentiation of the underlying mechanisms of hyponatremia, such as syndrome of inappropriate antidiuretic hormone secretion or cerebral salt wasting. In addition, information regarding peri-procedural IV fluid administration was not standardized and therefore could not be fully accounted for.

Fourth, the complete-case approach and exclusion of patients with incomplete laboratory or outcome data may have introduced selection bias. Furthermore, because variable-specific missingness among patients excluded during dataset construction could not be retrospectively reconstructed, the pattern and mechanism of missingness could not be fully characterized.

Fifth, given the exploratory evaluation of multiple clinical, laboratory, and peri-procedural electrolyte parameters without formal adjustment for multiple comparisons, the possibility of chance findings (Type I error) cannot be excluded. Although post-procedural serum Na remained independently associated with mortality in the multivariable analysis and this association was supported by bootstrap validation, the finding should be considered hypothesis-generating rather than confirmatory and requires validation in adequately powered prospective studies.

Sixth, the sample size of our cohort may have resulted in limited statistical power for certain variables. The post hoc analysis demonstrated an achieved power of 56.7% for the observed difference in post-procedural Na between the survival and mortality groups. Moreover, although pre-procedural Na approached statistical significance in the univariate analysis (*p* = 0.062), neither pre-procedural Na nor ΔNa was independently associated with mortality in the adjusted multivariable models. Therefore, the absence of statistically significant associations for these parameters should not be interpreted as definitive evidence of no effect, and the possibility of Type II error cannot be excluded. Larger cohorts are required to more precisely determine the potential clinical relevance of these electrolyte parameters.

Seventh, although the extended model including post-procedural serum Na showed no evidence of poor calibration, the addition of post-procedural serum Na to the established clinical, procedural, and metabolic predictors resulted in only a modest numerical increase in discrimination (AUC 0.800 to 0.812; ΔAUC = 0.012), which was not statistically significant according to DeLong’s test (*p* = 0.454). Therefore, despite its independent association with mortality, the present study does not establish significant incremental predictive utility of post-procedural serum Na beyond the predictors included in the base model. Further external validation in larger independent cohorts is required before its potential contribution to prognostic modelling can be established.

Finally, given the retrospective and observational design of this study, the observed relationships should be interpreted as associative rather than causal. Residual confounding cannot be completely excluded, and further prospective studies are required to determine whether Na disturbances are merely prognostic markers or whether their targeted correction can directly improve patient outcomes.

Despite these limitations, this study provides novel, hypothesis-generating evidence regarding the potential prognostic value of early post-procedural serum Na in patients undergoing MT.

## 5. Conclusions

In this study, it was demonstrated that the admission NIHSS score at presentation, successful reperfusion, and symptomatic intracranial hemorrhage are the strongest independent prognostic factors for 90-day mortality in patients who underwent MT for AIS. In addition, post-procedural serum Na levels were independently associated with 90-day mortality after adjustment for relevant clinical, procedural, and metabolic variables. However, the addition of post-procedural serum Na did not significantly improve model discrimination beyond these predictors. Therefore, although post-procedural serum Na may represent an associated prognostic marker, its incremental predictive utility remains unproven. Moreover, given the retrospective observational design, these findings should be considered hypothesis-generating and should not be interpreted as evidence of a causal effect of serum Na on 90-day mortality. In contrast, it was observed that pre-procedure electrolyte levels and peri-procedural electrolyte changes did not reach statistical significance in the adjusted models.

Our study is one of a limited number of studies that not only evaluate baseline electrolyte levels before and after mechanical thrombectomy in patients with AIS but also examine the prognostic value of peri-procedural electrolyte changes. The findings suggest that post-procedural serum Na levels, in particular, may contribute to early prognostic assessment as an easily accessible and routinely measured biomarker that may hypothetically relate to the pathophysiological processes developing after reperfusion. However, due to the observational study design, it cannot be concluded that the association between post-procedure serum Na levels and mortality is causal.

In conclusion, evaluating post-procedural serum Na levels in conjunction with existing clinical and imaging markers may warrant further evaluation as a prognostic marker in patients undergoing MT. However, larger, multicenter, prospective studies are needed to validate these findings, elucidate the underlying mechanisms, and to determine their role in clinical practice.

## Figures and Tables

**Table 1:** Baseline clinical and demographic characteristics according to outcome.

Variable	Total (*n* = 274)	Survival (mRS 0–5) (*n* = 207)	Mortality (mRS 6) (*n* = 67)	*p*-Value
Age (years)	68.77 ± 11.48	68.84 ± 11.42	68.63 ± 12.00	0.898
Female sex	146 (53.3%)	113 (54.6%)	33 (49.3%)	0.447
Current smoking	48 (17.5%)	38 (18.4%)	10 (14.9%)	0.521
Chronic alcohol use	18 (6.6%)	14 (6.8%)	4 (6.0%)	1.000
Hypertension	182 (66.4%)	135 (65.2%)	47 (70.1%)	0.457
Diabetes mellitus	66 (24.1%)	45 (21.7%)	21 (31.3%)	0.110
Obesity	45 (16.4%)	31 (15.0%)	14 (20.9%)	0.256
Dyslipidemia	128 (46.7%)	98 (47.3%)	30 (44.8%)	0.714
Atrial fibrillation	135 (49.3%)	106 (51.2%)	29 (43.3%)	0.259
Coronary artery disease	96 (35.0%)	67 (32.4%)	29 (43.3%)	0.104
Previous CABG	18 (6.6%)	10 (4.8%)	8 (11.9%)	**0.049**
Peripheral artery disease	7 (2.6%)	6 (2.9%)	1 (1.5%)	1.000
Current malignancy	14 (5.1%)	10 (4.8%)	4 (6.0%)	0.751
Previous stroke/TIA	59 (21.5%)	42 (20.3%)	17 (25.4%)	0.379

Continuous variables are presented as mean ± standard deviation (SD), and categorical variables as *n* (%). Bold *p*-values indicate statistical significance (*p* < 0.05). Abbreviations: SD, standard deviation; CABG, coronary artery bypass grafting; TIA, transient ischemic attack; mRS, modified Rankin Scale.

**Table 2:** Stroke etiology distribution according to outcome.

Variable	Total (*n* = 274)	Survival (mRS 0–5) (*n* = 207)	Mortality (mRS 6) (*n* = 67)	*p*-Value
Large artery atherosclerosis	42 (15.4%)	30 (14.6%)	12 (17.9%)	0.079
Cardioembolism	148 (54.2%)	117 (56.8%)	31 (46.3%)	
Other determined etiology	19 (7.0%)	10 (4.9%)	9 (13.4%)	
Undetermined etiology	64 (23.4%)	49 (23.8%)	15 (22.4%)	

Data are presented as *n* (%). The *p* value refers to the overall comparison of stroke etiology distribution between the survival and mortality groups. Abbreviation: mRS, modified Rankin Scale.

**Table 3:** Procedural and imaging characteristics according to outcome.

Variable	Total (*n* = 274)	Survival (mRS 0–5) (*n* = 207)	Mortality (mRS 6) (*n* = 67)	*p*-Value
Admission NIHSS score	17 (15–19)	17 (14–18)	19 (16–20)	**<0.001**
ASPECTS	9 (8–10)	9 (8–10)	9 (8–9)	**0.039**
Admission SBP, mmHg	153.6 ± 32.8	152.7 ± 33.0	156.5 ± 32.3	0.452
Admission DBP, mmHg	87.7 ± 17.7	87.4 ± 18.4	88.3 ± 15.7	0.672
Symptom onset-to-reperfusion time, min	245 (191–334)	239 (184–336)	251 (208–325)	0.305
Successful reperfusion (TICI 2b, 3)	239 (87.2%)	188 (90.8%)	51 (76.1%)	**0.001**
Intraprocedural intubation due to respiratory distress	8 (2.9%)	2 (1.0%)	6 (9.0%)	**0.003**
Symptomatic intracranial hemorrhage after EVT	38 (13.9%)	17 (8.2%)	21 (31.3%)	**<0.001**
EVT-related complication	19 (6.9%)	14 (6.7%)	5 (7.5%)	0.788

Continuous variables are presented as mean ± SD or median (IQR), as appropriate; categorical variables are presented as *n* (%). Bold *p*-values indicate statistical significance (*p* < 0.05). Among EVT-related complications, vessel rupture was not observed in either group. Dissection occurred in 3 patients in the survival group and none in the mortality group, reocclusion occurred in 1 patient in the mortality group and none in the survival group, and vasospasm was observed in 11 patients in the survival group and 4 patients in the mortality group. Abbreviations: SBP, systolic blood pressure, DBP, diastolic blood pressure; NIHSS, National Institutes of Health Stroke Scale; IQR, interquartile range; SD, standard deviation; EVT, endovascular thrombectomy; TICI, Thrombolysis in Cerebral Infarction; mRS, modified Rankin Scale.

**Table 4:** Hematologic and biochemical parameters according to outcome.

Variable	Total (*n* = 274)	Survival (mRS 0–5) (*n* = 207)	Mortality (mRS 6) (*n* = 67)	*p*-Value
Hemoglobin, g/dL	12.67 ± 1.93	12.71 ± 1.86	12.49 ± 2.15	0.430
Hematocrit, %	38.70 (34.65–42.90)	38.9 (34.8–42.9)	37.7 (33.3–42.9)	0.431
Leukocyte count,/µL	8540 (6830–10,400)	8480 (6630–10,400)	8950 (7380–10,110)	0.211
Platelet count, ×10^3^/µL	211 (169–258)	211 (170–258)	212 (168–256)	0.764
Glucose, mg/dL	129 (106–171)	123.0 (103.0–151.0)	164.0 (124.0–201.0)	**<0.001**
Total cholesterol, mg/dL	174.68 ± 42.64	174.5 ± 41.9	176.9 ± 44.6	0.685
LDL-C, mg/dL	115.02 ± 33.17	114.8 ± 32.9	116.7 ± 33.7	0.688
HDL-C, mg/dL	43.57 ± 11.03	44.2 ± 10.4	42.1 ± 12.6	0.172
Triglycerides, mg/dL	78 (57–115)	73.0 (56.0–110.0)	99.5 (70.0–131.0)	**0.001**
Albumin, g/dL	3.69 (3.39–3.90)	3.69 (3.40–3.89)	3.76 (3.33–3.90)	0.530

Continuous variables are presented as mean ± SD or median (IQR), as appropriate. Bold *p*-values indicate statistical significance (*p* < 0.05). Abbreviations: SD, standard deviation; IQR, interquartile range; LDL-C, low density lipoprotein cholesterol; HDL-C, high density lipoprotein cholesterol; mRS, modified Rankin Scale.

**Table 5:** Peri-procedural electrolyte parameters according to outcome.

Variable	Total (*n* = 274)	Survival (mRS 0–5) (*n* = 207)	Mortality (mRS 6) (*n* = 67)	*p*-Value
Pre-procedural Na^+^, mmol/L	139.0 (137.0–140.0)	139.0 (137.0–141.0)	138.0 (136.0–140.0)	0.062
Post-procedural Na^+^, mmol/L	136.16 ± 2.64	136.36 ± 2.56	135.55 ± 2.83	**0.030**
ΔNa^+^, mmol/L	−2.0 (−4.0 to −1.0)	−2.0 (−4.0 to −0.8)	−2.0 (−4.0 to −1.0)	0.959
Pre-procedural K^+^, mmol/L	4.2 (3.9–4.4)	4.2 (3.9–4.4)	4.2 (3.9–4.5)	0.479
Post-procedural K^+^, mmol/L	4.1 (3.9–4.4)	4.1 (3.9–4.4)	4.1 (3.9–4.5)	0.585
ΔK^+^, mmol/L	0.0 (−0.3 to 0.2)	0.0 (−0.3 to 0.2)	0.0 (−0.3 to 0.2)	0.515
Pre-procedural Cl^−^, mmol/L	104.0 (102.0–106.0)	104.0 (102.0–106.0)	104.0 (101.0–105.0)	0.051
Post-procedural Cl^−^, mmol/L	104.0 (101.0–106.0)	104.0 (101.0–106.0)	103.0 (101.0–105.0)	0.389
ΔCl^−^, mmol/L	0.0 (−2.0 to 1.0)	0.0 (−2.0 to 1.0)	0.0 (−2.0 to 1.0)	0.410
Pre-procedural Ca^2+^, mg/dL	9.2 (8.9–9.6)	9.2 (8.9–9.6)	9.1 (8.8–9.6)	0.611
Post-procedural Ca^2+^, mg/dL	8.6 (8.3–8.9)	8.6 (8.4–9.0)	8.6 (8.1–8.8)	0.229
ΔCa^2+^, mg/dL	−0.6 (−0.9 to −0.3)	−0.6 (−0.8 to −0.3)	−0.7 (−1.0 to −0.2)	0.283

Continuous variables are presented as median (IQR). Δ values were calculated as post-procedural minus preprocedural levels. Bold *p*-values indicate statistical significance (*p* < 0.05). Abbreviations: IQR, interquartile range; Na, sodium; K, potassium; Cl, chloride; Ca, calcium; SD, standard deviation; mRS, modified Rankin Scale.

**Table 6:** Univariate Logistic Regression Analysis for Factors Associated with Mortality.

Variable	OR (95% CI)	*p*-Value
Coronary artery disease	1.595 (0.907–2.803)	0.105
Previous CABG	2.671 (1.008–7.076)	**0.049**
Admission NIHSS (per 1-point increase)	1.199 (1.095–1.313)	**<0.001**
ASPECTS (per 1-point increase)	0.772 (0.592–1.008)	0.058
Successful reperfusion (TICI 2b, 3)	0.288 (0.136–0.610)	**0.001**
Intubation due to respiratory distress during procedure	10.033 (1.974–50.984)	**0.003**
Symptomatic intracranial hemorrhage after EVT	5.102 (2.494–10.440)	**<0.001**
Glucose (per 1 mg/dL increase)	1.008 (1.004–1.012)	**<0.001**
Triglycerides (per 10 mg/dL increase)	1.050 (1.013–1.089)	**0.001**
Pre-procedural sodium (per 1 mmol/L increase)	0.916 (0.836–1.004)	0.062
Post-procedural sodium (per 1 mmol/L increase)	0.890 (0.800–0.990)	**0.031**
ΔNa (per 1 mmol/L increase)	0.996 (0.892–1.111)	0.939
Pre-procedural potassium (per 1 mmol/L increase)	1.598 (0.892–2.865)	0.115
Post-procedural potassium (per 1 mmol/L increase)	1.252 (0.675–2.322)	0.475
ΔK (per 1 mmol/L increase)	0.653 (0.313–1.360)	0.255
Pre-procedural chloride (per 1 mmol/L increase)	0.930 (0.866–0.998)	0.051
Post-procedural chloride (per 1 mmol/L increase)	0.967 (0.896–1.044)	0.393
ΔCl (per 1 mmol/L increase)	1.086 (0.985–1.197)	0.410
Pre-procedural calcium (per 1 mg/dL increase)	0.832 (0.524–1.321)	0.435
Post-procedural calcium (per 1 mg/dL increase)	0.636 (0.366–1.107)	0.109
ΔCa (per 1 mg/dL increase)	0.839 (0.500–1.410)	0.508

Odds ratios (ORs) and 95% confidence intervals (CIs) were obtained from univariate logistic regression analyses with mortality as the dependent variable. Continuous variables were entered per unit increase as indicated in the table. ORs for continuous variables are presented per 1-unit increase, except for triglycerides, which are presented per 10 mg/dL increase. This specific scaling was chosen a priori to provide a clinically interpretable effect size, as a 1 mg/dL fluctuation in triglycerides is not clinically perceptible and yields mathematically marginal OR values. Bold *p*-values indicate statistical significance (*p* < 0.05). Abbreviations: OR, odds ratio; CI, confidence interval; NIHSS, National Institutes of Health Stroke Scale; TICI, Thrombolysis in Cerebral Infarction; EVT, endovascular thrombectomy; CABG, coronary artery bypass grafting.

**Table 7:** Multivariable logistic regression models for mortality.

Variable	Model 1: Pre-Procedural Na OR (95% CI)	*p*	Model 2: Post-Procedural Na OR (95% CI)	*p*	Model 3: ΔNa OR (95% CI)	*p*
Admission NIHSS (per 1-point increase)	1.188 (1.075–1.313)	**0.001**	1.178 (1.065–1.302)	**0.001**	1.176 (1.064–1.300)	**0.001**
Successful reperfusion (TICI 2b, 3)	0.315 (0.129–0.770)	**0.011**	0.304 (0.122–0.757)	**0.011**	0.329 (0.134–0.804)	**0.012**
Intubation due to respiratory distress during procedure	6.847 (1.110–42.240)	**0.038**	7.063 (1.124–44.370)	**0.037**	6.627 (1.041–42.207)	**0.042**
Symptomatic intracranial hemorrhage after EVT	5.044 (2.225–11.438)	**<0.001**	5.706 (2.455–13.262)	**<0.001**	5.348 (2.365–12.094)	**<0.001**
Glucose (per 1 mg/dL increase)	1.006 (1.001–1.010)	**0.010**	1.007 (1.002–1.011)	**0.003**	1.007 (1.003–1.012)	**0.002**
Triglycerides (per 10 mg/dL increase)	1.048 (1.004–1.095)	**0.032**	1.045 (1.001–1.092)	**0.046**	1.049 (1.005–1.095)	**0.025**
Symptom onset-to-reperfusion time (per 1 min increase)	1.000 (0.998–1.002)	0.934	1.000 (0.998–1.002)	0.873	1.000 (0.998–1.002)	0.932
Pre-procedural Na (per 1 mmol/L increase)	0.917 (0.821–1.024)	0.124				
Post-procedural Na (per 1 mmol/L increase)			0.851 (0.749–0.968)	**0.014**		
ΔNa (per 1 mmol/L increase)					0.939 (0.819–1.076)	0.364

All three models were adjusted for admission NIHSS, successful reperfusion (TICI 2b, 3), intraprocedural intubation due to respiratory distress, symptomatic intracranial hemorrhage after EVT, admission glucose, triglyceride levels, and symptom onset-to-reperfusion time. Pre-procedural sodium, post-procedural sodium, and Δ sodium were evaluated in separate models because Δ sodium is mathematically derived from the pre- and post-procedural measurements (Δ sodium = post-procedural sodium − pre-procedural sodium), and simultaneous inclusion could introduce collinearity. ORs for continuous variables are presented per 1-unit increase, except for triglycerides, for which the OR is presented per 10 mg/dL increase to facilitate clinical interpretation. Bold *p*-values indicate statistical significance (*p* < 0.05). Abbreviations: OR, odds ratio; CI, confidence interval; EVT, endovascular thrombectomy; NIHSS, National Institutes of Health Stroke Scale; TICI, Thrombolysis in Cerebral Infarction; Na, sodium.

**Table 8:** Bootstrap validation of the multivariable logistic regression models for 90-day mortality.

Variable	OR (95% CI)	*p*-Value	Bootstrap *p*-Value	BCa 95% CI for B
Model 1: Pre-procedural Na	0.917 (0.821–1.024)	0.124	0.133	−0.212 to 0.038
Model 2: Post-procedural Na	0.851 (0.749–0.968)	**0.014**	**0.023**	**−0.312 to −0.045**
Model 3: ΔNa	0.939 (0.819–1.076)	0.364	0.389	−0.213 to 0.062

Model stability was assessed using nonparametric bootstrap resampling with 2000 samples. Bootstrap *p*-values and bias-corrected and accelerated (BCa) 95% confidence intervals are presented for the regression coefficient (B) of the sodium parameter evaluated in each model. Pre-procedural Na, post-procedural Na, and ΔNa were evaluated separately in Models 1, 2, and 3, respectively. Bold *p*-values indicate statistical significance (*p* < 0.05). Abbreviations: OR, odds ratio; CI, confidence interval; BCa, bias-corrected and accelerated; B, regression coefficient; Na, sodium; ΔNa, peri-procedural change in serum sodium (post-procedural Na − pre-procedural Na).

## Data Availability

The data presented in this study are available upon reasonable request from the corresponding author. The data are not publicly available due to privacy and ethical restrictions.

## Abbreviations

The following abbreviations are used in this manuscript:

AIS	Acute Ischemic Stroke
ASPECTS	Alberta Stroke Programme Early CT Score
AF	Atrial Fibrillation
Bca	Bias-corrected and accelerated
CABG	Coronary Artery Bypass Grafting
CAD	Coronary Artery Disease
Ca	Calcium
CI	Confidence Interval
Cl	Chloride
CT	Computed Tomography
DM	Diabetes Mellitus
DBP	Diastolic Blood Pressure
EVT	Endovascular Thrombectomy
HT	Hypertension
HDL-C	High-Density Lipoprotein Cholesterol
ICU	Intensive Care Unit
IQR	Interquartile Range
IV	Intravenous
K	Potassium
LDL-C	Low-Density Lipoprotein Cholesterol
LVO	Large Vessel Occlusion
mRS	Modified Rankin Scale
MT	Mechanical Thrombectomy
Na	Sodium
NIHSS	National Institutes of Health Stroke Scale
ROC	Receiver Operating Characteristic
OR	Odds Ratio
PAD	Peripheral Artery Disease
Q–Q	Quantile–Quantile
SBP	Systolic Blood Pressure
SD	Standard Deviation
TIA	Transient Ischemic Attack
TICI	Thrombolysis in Cerebral Infarction
TOAST	Trial of Org 10172 in Acute Stroke Treatment
Δ	Delta [Peri-procedural Change (Post-procedural − Pre-procedural)]
